# Purin Metabolism Is Crucial for Regulatory T Cell Stability and Function

**DOI:** 10.1002/eji.70070

**Published:** 2025-10

**Authors:** Young S. Lee, Marina W. Shirkey, Vikas Saxena, Dejun Kong, Bing Ma, Reza Abdi, Jonathan S. Bromberg

**Affiliations:** 1 Department of Surgery, University of Maryland School of Medicine, Baltimore, Maryland, USA; 2 Center For Vascular and Inflammatory Diseases, University of Maryland School of Medicine, Baltimore, Maryland, USA; 3 Institute For Genome Sciences, University of Maryland School of Medicine, Baltimore, Maryland, USA; 4 Department of Microbiology and Immunology, University of Maryland School of Medicine, Baltimore, Maryland, USA; 5 Transplantation Research Center, Renal Division, Harvard Medical School, Brigham and Women’s Hospital, Boston, Massachusetts, USA

**Keywords:** exTregs, purine metabolism, purinergic receptors, regulatory T cells (Tregs), Treg stability

## Abstract

Cellular metabolism intricately directs the differentiation, stability, and function of regulatory T cells (Tregs), which are pivotal in immune regulation. Metabolic reprogramming enables Tregs to adapt to diverse tissue environments; however, it can also disturb immune equilibrium, driving their conversion into unfavorable states like exTregs that hinder regulation in autoimmunity and transplantation. Purine metabolism has emerged as a critical but underexplored regulator of Treg biology. Beyond their traditional roles in nucleotide synthesis and energy balance, purine metabolites also serve as potent second messengers shaping Treg phenotype, suppressive capacity, and adaptability in inflammatory, autoimmune, and transplant environments. Extracellular ATP promotes inflammation, while adenosine supports Treg-mediated immunosuppression, highlighting a dual and context-dependent nature of purinergic signaling. This review outlines current findings on intracellular and extracellular purine metabolism in Tregs, emphasizing key enzymes and purinergic receptors that sustain Treg phenotype and resilience. It discusses disruptions in purine signaling compromising Treg functions, identifies knowledge gaps, and proposes future research directions for potential therapeutic strategies in immune-related ailments.

## 1 | Introduction: Metabolic Regulation of Regulatory T Cell Stability and Function

Regulatory T cells (Tregs), identified by the expression of Forkhead box protein 3 (Foxp3), maintain immune homeostasis and prevent autoimmunity. Their stability and function are regulated by a network of transcriptional, epigenetic, and posttranslational mechanisms, many of which are directly driven by cellular metabolism [1–3]. Metabolic reprogramming is not only a consequence of Treg activation but also a key determinant of their fate, function, and adaptability in diverse microenvironments [4].

Tregs exhibit metabolic heterogeneity depending on activation and functional state. Proliferative Tregs preferentially rely on fatty acid oxidation (FAO) over glycolysis to sustain long-term suppression capacity. Conversely, glycolysis is essential for migration and expansion. Mitochondrial metabolism supports their suppressive function. Disrupting these metabolic pathways impairs Treg differentiation and behavior [5–7], potentially causing instability, impairing their suppressive function, and promoting conversion into exTregs (Foxp3^lo^CD25^lo^), which acquire proinflammatory properties and contribute to immune dysregulation [8, 9].

While glucose and lipid metabolism are well-established regulators of Tregs, purine metabolism is also a crucial but underexplored modulator of immune function. Purines, including ATP and its metabolites, regulate proliferation, migration, apoptosis, and immune functions. In Tregs, extracellular purines engage purinergic receptors and are modulated by ectonucleotidases CD39 and CD73, which regulate the ATP-adenosine balance and influence immunosuppressive activity [10–12]. Recent research links purinergic signaling to lymphotoxin-mediated Treg migration and function, affecting Treg stability by altering conversion to exTregs and influencing allograft survival [6].

This review explores the role of purinergic metabolism in Tregs, addressing (1) how purines regulate Treg stability and function; (2) the roles of purinergic receptors in these processes; and (3) enzymes and transporters controlling purine homeostasis. We also discuss disruptions in purinergic pathways that compromise Treg functions, identify knowledge gaps, and suggest future research directions for developing potential therapeutic strategies.

### 1.1 | Purine Metabolites Influence Treg Expansion and Function

**FIGURE 1 |:**
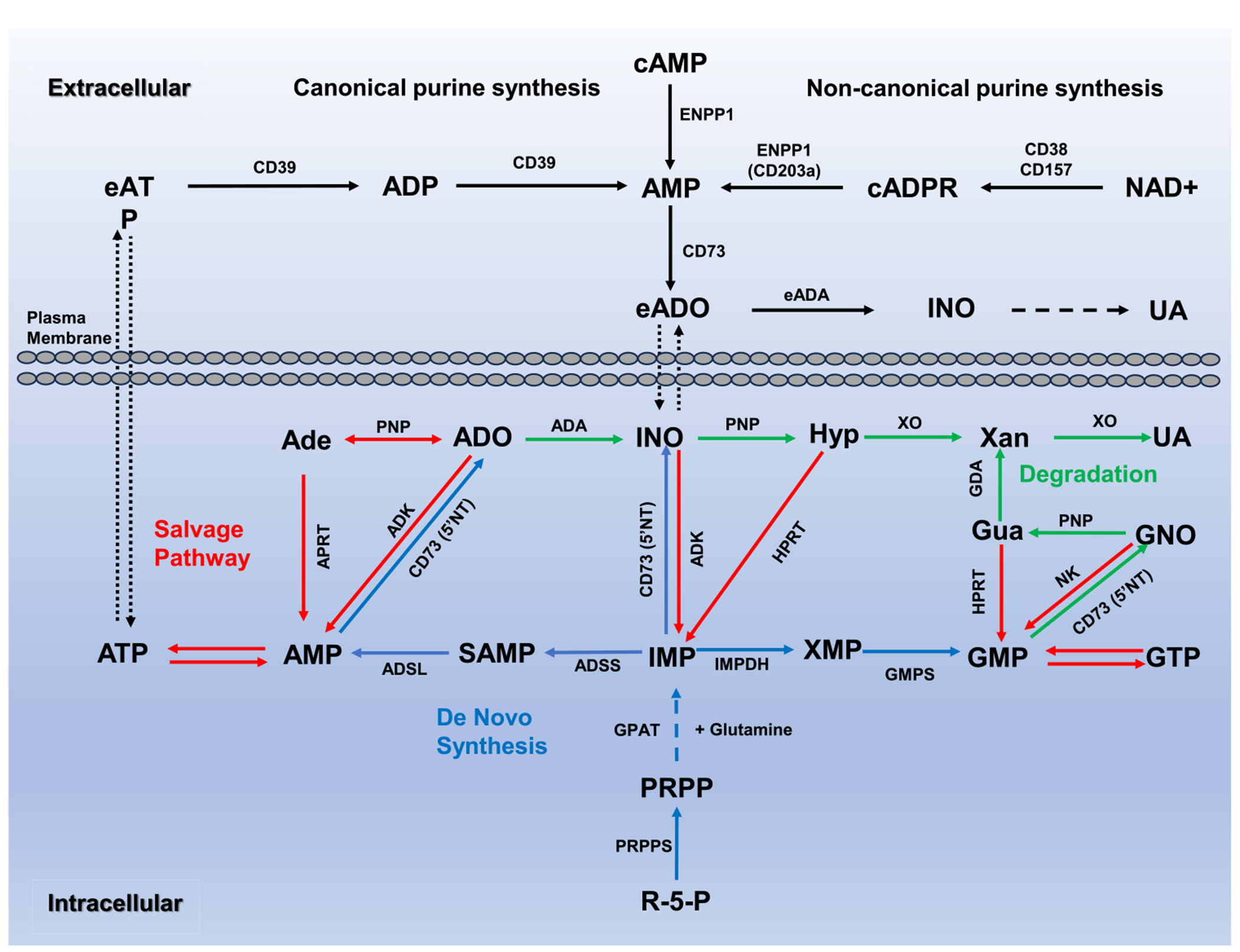
An overview of purine metabolism. Purine metabolism is tightly regulated by interconnected anabolic and catabolic pathways, encompassing both intracellular and extracellular processes. De novo synthesis initiates with ribose-5-phosphate (R-5-P) and progresses through enzymatic steps involving PRPP synthetase (PRPPS) and amidophosphoribosyltransferase (GPAT), ultimately generating inosine monophosphate (IMP). The salvage pathway recycles purine bases, including adenine (Ade), guanine (Gua), and hypoxanthine (Hyp), via adenine phosphoribosyltransferase (APRT) and hypoxanthine-guanine phosphoribosyltransferase (HPRT), forming AMP, GMP, and IMP. Purine catabolism leads to uric acid (UA) through purine nucleoside phosphorylase (PNP) and xanthine oxidase (XO). Extracellular purine metabolism involves catabolism of extracellular ATP (eATP) by membrane-bound ectonucleotidases. CD39 (ENPDase) converts eATP to AMP, which CD73 (5’NT) further hydrolyzes extracellular adenosine (eADO). Ecto-adenosine deaminase (eADA) subsequently metabolizes eADO into inosine (INO). Noncanonical purine metabolism utilizes extracellular nicotinamide adenine dinucleotide (NAD+) to generate AMP and subsequently ADO. Ectoenzymes CD38 and CD157 convert NAD+ to cyclic ADP-ribose (cADPR), which is converted to AMP by ectonucleotide phosphohydrolase/phosphodiesterase 1 (ENPP1 or CD203a) and further hydrolyzed to ADO by CD73.

Purines, beyond their canonical roles in nucleic acid synthesis and energy production, act as signaling molecules that regulate cellular behaviors and immune responses. Purinergic signaling, primarily driven by adenosine triphosphate (ATP) and adenosine (ADO), regulates Treg activation, stability, and cytokine production. These metabolites are delicately regulated through intracellular de novo and salvage pathways, and extracellular ATP- and NAD+-metabolizing pathways involving key enzymes (Figure 1).

### 1.2 | Extracellular ATP: Dual Roles in Treg Stability and Function

Extracellular ATP (eATP), released by dying, cancer, and immune or stromal cells, exerts context-dependent effects on Tregs, with its distinct impact depending on concentration, receptor engagement, and disease context. eATP impairs Treg function and stability by promoting proinflammatory responses and inhibiting their suppressive capabilities. Schenk et al. [13] demonstrate that ATP downregulates Foxp3 expression in Tregs in vivo, converting them into IL-17-secreting proinflammatory cells and impairing their suppressive function. They also show that ATP activates the P2×7 receptor (P2×7R), leading to increased ERK phosphorylation, which further destabilizes Tregs, as evidenced by increased Foxp3 expression when ERK is inhibited with the pharmacologic inhibitor PD98059. eATP inhibits Treg development while promoting Th17 cell differentiation, worsening T cell-mediated colitis in mice [14] and obesity-induced inflammation in humans [15]. In colitis, control mice have lower intestinal ATP and fewer lamina, which increase Th17 cell numbers and induce Th17-promoting molecules like IL-6, IL-23, and integrins *α*V and *β*8 [14]. In obesity, ATP stimulates the P2×7R, creating a Th17-polarizing microenvironment propria Th17 cells. Intraperitoneal administration of ATP to control mice every 3 days for 15 days elevates IL-1*β*, IL-6, and IL-17 levels in human visceral adipose tissue from obese patients [15]. In wild-type Tregs stimulated with CD3, IL-6 increases extracellular ATP levels and phosphorylation of ERK, and reduces Foxp3 expression compared with CD3 stimulation alone [13]. In contrast, in *P2rx7*^−/−^ Tregs, IL-6 reduces ATP levels without affecting Foxp3 expression. These data indicate that IL-6 induces ATP-P2×7R signaling and impairs Treg stability via activation of mitogen-activated protein kinase (MAPK), potentially via increased intracellular Ca^2+^ influx, a process facilitated when eATP binds to P2X receptor cation channels.

In contrast, eATP supports Treg proliferation and suppressive function under certain conditions. Lecciso et al. [16] observe that increased ATP release associates with increased numbers of PD-1-expressing suppressive Tregs in both human acute myeloid leukemia (AML) patients undergoing chemotherapy with daunorubicin (DNR) and cytarabine (ARA-C) and in mice treated with DNR or ARA-C. This effect is absent in mice lacking P2×7R. Another study shows that eATP exerts opposing effects on activated and regulatory CD4^+^ T cells, depending on its concentration. Using anti-CD3/CD28-activated CD4^+^ T cells and Tregs from healthy human PBMCs, high ATP levels (1 mM) enhance Treg proliferation and suppressive function via the P2Y2 receptor (P2Y2R), while inducing apoptosis in Teff cells through P2×7R and P2×4R [17]. At lower physiological ATP levels (1–50 nM), neither effector T cells (Teffs) nor Tregs were significantly affected, indicating threshold-dependent effects of ATP. To integrate and clarify these findings on the dual and context-dependent effects of eATP on Tregs, Table 1 summarizes the experimental or pathological conditions, corresponding outcomes related to Treg enhancement or suppression, the receptors and signaling pathways involved, and the implications for tissue-specific immunoregulation and translational strategies targeting purinergic signaling.

### 1.3 | Extracellular Adenosine: A Central Immunoregulatory Signal in Tregs

Extracellular adenosine (eADO) is primarily produced from the breakdown of ATP, ADP, and AMP through sequential enzymatic hydrolysis by CD39 (ecto-NTPDase-1) and CD73 (ecto-5′-nucleotidase). Tregs express high levels of these ectoenymes, enabling them to hydrolyze eATP and generate eADO, unlike resting conventional T cells. In mice, Tregs hydrolyze eATP to generate eADO, which activates adenosine A2A receptor (A2AR) on Teffs and suppresses their proliferation and function [18]. CD39-deficient Tregs show impaired suppressive function and fail to prevent skin allograft rejection. In both mice and humans, Treg-produced eADO suppresses CD8+ T cell-mediated antitumor immunity, contributing to immunosuppression in tumors [19]. In patients with head and neck squamous cell carcinoma (HNSCC), Tregs have elevated CD39 and CD73 [20], enabling more efficient ATP hydrolysis and eADO production [21]. This suppression can be blocked by ectonucleotidase inhibitors or an A2AR antagonist.

eADO binds to the A2AR and increases cyclic adenosine monophosphate (cAMP) levels in Tregs [22, 23]. Increased cAMP stabilizes Foxp3 expression, increases CD39 mRNA expression, promotes CD39 and CD73 activity, and increases ADO production, which forms an ADO-cAMP feedback loop that strengthens Treg suppressive function, as shown in an experimental autoimmune uveitis (EAU) mouse model [23]. Inhibition of CD39 or CD73, using either specific siRNAs or pharmacological inhibitors, such as sodium polyoxotungstate (POM-1) for CD39 and (adenosine 5′-(*α*,*β*-methylene) diphosphate (APCP) for CD73, decreases ADO levels and inhibits Treg-mediated suppression.

ADO stimulates A2AR, exerting a distinct effect on Tregs and effector T cells, as shown in an autoimmune pneumonitis mouse model [24]. In Tregs, tissue-derived ADO-A2AR signaling promotes proliferation, enhances suppressive function, and increases Foxp3 expression. In effector T cells, ADO stimulation upregulates A2AR, increases cAMP levels, but induces an anergy-like hyporesponsive state, and suppresses Th1 and Th17 activity. This dual action of ADO is essential for maintaining immune balance; transfer of wild-type T cells fosters tolerance and ensures 100% survival in pneumonitis-induced mice. However, transfer of A2AR^−/−^ T cells disrupts this balance, resulting in 80% mortality due to the loss of ADO-mediated tolerizing effects and subsequent tissue destruction.

ADO accumulation in the tumor microenvironment (TME), driven by the overexpression of CD39 and CD73 on cancer cells, stromal cells, and Tregs, is a hallmark of immunosuppression that promotes tumor progression. High ADO levels enhance Treg expansion, stability, and immunosuppressive potential, while also inducing metabolic reprogramming. It promotes glycolysis to meet energy demands for migration [25] and facilitates lactic acid production, which further supports Treg function and suppresses effector T cells by acidifying the TME [26]. Simultaneously, A2AR signaling shifts Treg metabolism toward oxidative phosphorylation (OXPHOS). This transition enables Tregs to utilize alternative energy sources, such as fatty acids and lactate, which are abundantly available in the TME due to tumor and stromal cell metabolism. OXPHOS supports the long-term survival and sustained suppressive function of Tregs by providing a steady supply of ATP and reducing the reliance on glucose, which is often scarce in the TME. ADO promotes FAO, a vital energy source under oxidative stress, as demonstrated in human ovarian cancer and mouse colorectal cancers [21, 31]. This metabolic adaptation equips Tregs to thrive in nutrient-depleted conditions typical of the TME. ADO also induces lipid synthesis to support membrane biosynthesis and signaling required for Treg proliferation and suppression [7, 27]. This ADO-mediated regulation of lipid metabolism ensures that Tregs can balance energy production with the physiologic demands of rapid expansion within the TME.

ADO signaling in Tregs enhances the production of immunosuppressive cytokines such as interleukin-10 (IL-10) and transforming growth factor-beta (TGF-*β*) [28]. These cytokines suppress Teffs and other immune cells, fostering an immunosuppressive tumor environment, limiting the development of inflammatory diseases and chronic infections, reducing the risk of autoimmune diseases, and promoting tolerance to allogeneic grafts [19, 29]. IL-10 inhibits proinflammatory cytokine production across multiple immune cell types (DCs, macrophages, mast cells, Th1/Th2 cells, Teffs, NK cells, and plasma cells) via the JAK-STAT signaling pathway [30] and promotes Treg differentiation through a positive feedback loop, maintaining their phenotype and function [31]. Similarly, TGF-*β* promotes the generation of Foxp3^+^ Tregs, immunosuppressive M2 macrophages, and N2 neutrophils, and tolerogenic dendritic cells, reinforcing immune suppression [32–35].

eADO also serves as a vital regulator of intestinal immune balance, profoundly influencing Treg-driven tolerance in the mucosa. Microbiota-derived purine metabolism plays a critical role in shaping the extracellular nucleotide landscape of the intestinal microenvironment and directly influences the metabolic and functional stability of Tregs. Gut-resident Tregs are uniquely enriched in TCRs specific for commensal antigens, distinguishing them from Tregs in other tissues and highlighting their tight dependence on microbial cues for development and maintenance [36]. A recent study revealed that bacterial degradation of host- and diet-derived purines significantly reduces extracellular adenosine levels in the gut, thereby limiting local A2A receptor-mediated immunoregulation [37], potentially impairing Treg function. These findings suggest that fluctuations in microbial purine metabolism can alter Treg plasticity and stability within the intestinal niche. Therefore, microbial modulation of purine availability should be critically considered when evaluating the role of extracellular adenosine in gut immune tolerance and in designing therapeutic strategies targeting mucosal immunity.

High levels of ADO production and elevated expression of CD39 and CD73 in Tregs [38–40] are also observed in chronic viral infections such as HIV and COVID-19. CD39^+^ Tregs more effectively suppress CD4^+^ T cell function compared with CD39^−^ Tregs, and suppression is reversible by targeting CD39 or A2AR [39]. In COVID-19, the frequency of CD39^+^ Tregs correlates with disease severity, suggesting a role for purine signaling in sustaining immune dysfunction [41]. Targeting ADO pathways may represent a therapeutic strategy for restoring immune balance in chronic viral infections.

### 1.4 | Direct Mechanistic Links Between Purine Metabolites and Foxp3 Regulation

Although direct mechanistic evidence linking purine metabolites to Foxp3 transcription or translation is limited in currently available literature, a few notable studies provide relevant insights. In a recent sepsis study [42], extracellular adenosine was shown to promote Foxp3 expression in Tregs via A2A receptor engagement and downstream activation of the CREB transcription factor. Similarly, an earlier study by Bao et al. [43] demonstrated that adenosine and its analogs enhanced Foxp3 expression through JNK/AP-1 signaling, which could be inhibited by adenosine receptor antagonists or JNK blockade. These findings support the idea that adenosine can directly upregulate Foxp3 transcription under inflammatory conditions. Additionally, purine metabolism-related cofactors such as NAD^+^ have been implicated in supporting Foxp3 transcriptional activity and protein stability through epigenetic regulation, acting as essential substrates for chromatin-modifying enzymes [1].

### 1.5 | Purinergic Receptors Regulate Treg Function

**FIGURE 2 |:**
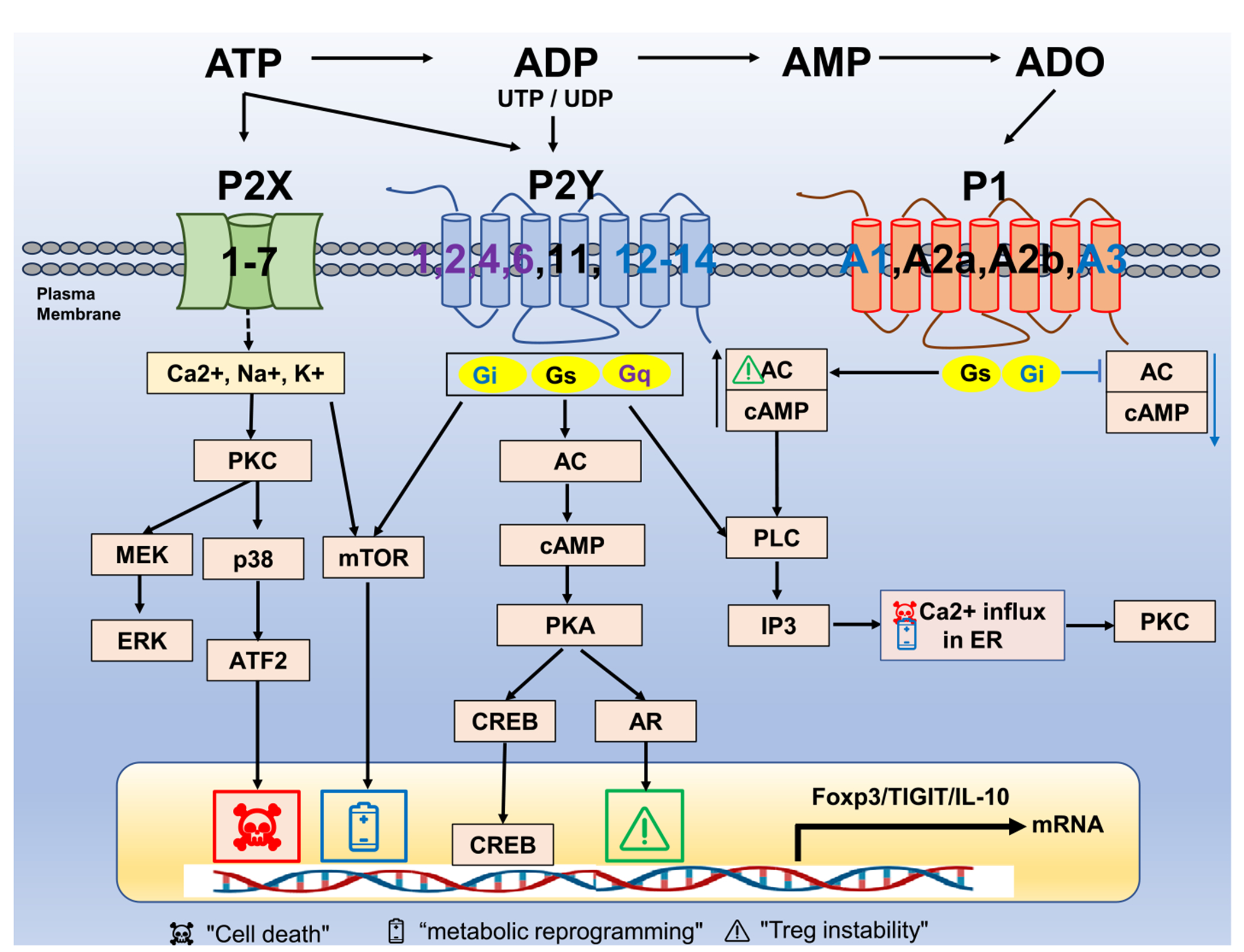
A schematic of purinergic receptors and key signaling in Tregs. Purine receptors regulate Treg stability and function by modulating intracellular signaling. These receptors fall into three subfamilies: P1, P2X, and P2Y. P1 receptors are G protein-coupled receptors (GPCRs) with A1, A2A, A2B, and A3 subtypes. A1 and A3 signal via Gi, inhibiting adenylate cyclase (AC), reducing cyclic adenosine monophosphate (cAMP) levels. A2A and A2B signal via Gs, activating AC, increasing cAMP. A2B also engages Gq, activating phospholipase C (PLC) and increasing intracellular calcium (Ca2+), influencing protein kinase C (PKC) signaling. P2Y receptors are GPCRs with eight subtypes (P2Y1, P2Y2, P2Y4, P2Y6, P2Y11, P2Y12, P2RY13, and P2Y14), responsive to ATP, UTP, ADP, and UDP with varying potencies (Table 2). Most (P2Y1, P2Y2, P2Y4, P2Y6, and P2Y11) signal via Gq, activating PLC, increasing Ca2+, and simulating PKC. P2Y12, P2Y13, and P2Y14 signal via Gi, inhibiting AC, reducing cAMP, and suppressing protein kinase A (PKA) signaling. P2Y11 can also act via Gs, activating AC and increasing cAMP. P2X receptors are ATP-gated ion channels permeable for Ca2+, Na+, and K+, contributing to Treg responses triggered by ATP binding. The symbolic icons are used to indicate the functional outcomes of signaling pathways: ☠ denotes associations with apoptosis and cellular stress (cell death), 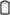 indicates involvement in metabolic reprogramming, and ⚠ highlights pathways known to disrupt Foxp3 expression or Treg stability (Treg instability).

Purinergic receptors regulate Treg function by sensing extracellular purine metabolites and modulating key regulatory pathways (Figure 2). They respond to dynamic levels of eATP and eADO, which elicit context-dependent effects [44]. Purinergic receptors are categorized into two subfamilies: P1, activated by ADO, and P2, which respond to ATP, ADP, UTP, and UDP.

P1 receptors are G protein-coupled receptors (GPCRs) that respond to eADO and consist of four subtypes: A1, A2A, A2B, and A3 receptors. These receptors regulate intracellular cAMP levels through their coupling to adenylate cyclase (AC) activity (Figure 2). A1 and A3 receptors couple with Gi proteins, inhibiting AC and resulting in decreased cAMP levels. Both receptors exhibit low ADO affinity, though A1R contributes to Tregs immunosuppressive function [45]. A2A and A2B receptors couple with Gs proteins, increasing AC activity and cAMP. A2AR, which has a high affinity for ADO, is highly expressed in Tregs [46], while A2B receptor (A2BR) has a lower affinity and less impact on cAMP regulation.

A1R and A2AR are the primary functional ADO receptors in Tregs. Tregs produce eADO, which suppresses effector T cell activation via A2AR-dependent signaling [47]. In healthy individuals, A2AR activation upregulates T-cell immunoglobulin and ITIM domain (TIGIT) expression in Tregs, and this induction is absent in autoimmune patients [47]. In mice, TIGIT^+^ Tregs are observed in lymphoid tissues and contribute to suppression of experimental autoimmune uveitis (EAU). The presence of A2AR in Tregs is critical for the development of these immunosuppressive TIGIT^+^ Tregs, as A2A-deficient mice exhibit impaired TIGIT^+^ Treg formation. Activation of A1R and A2AR enhances Treg expansion and triggers cAMP–CREB (cAMP response element-binding protein) and cAMP-AR (androgen receptor) signaling pathways, which increase IL-10 and Foxp3 expression [28]. Ohta et al. [48] demonstrated that A2AR agonists such as CGS21680 or NECA expand CD25^hi^Foxp3^+^ Tregs, which express CD39, CD73, and CTLA-4. A2AR signaling increases both the number and suppressive function of CD4^+^ CD25^+^ natural Tregs and induced Tregs (iTregs) derived from CD4^+^ CD25^−^ cells. Tregs lacking A2AR or treated with A2AR antagonists have reduced immunosuppressive capacity against Teffs [18, 49, 50].

The A2BR also contributes to Treg-mediated immunoregulation. In experimental autoimmune myositis, A2BR activation decreases Th17 cell frequency, increases Treg numbers, and prevents Treg exhaustion [51]. In a mouse model of lung ischemia-reperfusion injury (LIRI), A2BR blockade using a neutralizing antibody significantly reduces the Treg proportion in cultured peripheral blood from LIRI mice [52]. A2BR-deficient mice subjected to endotoxin-induced pulmonary inflammation show impaired Treg induction, enhanced recruitment of proinflammatory T cells, and exacerbated inflammation with increased fluid leakage into the airways [53].

P2 receptors consist of two subfamilies: P2X receptors (ATP-gated ion channels) and P2Y receptors (GPCRs). Both subfamilies play pivotal roles in ATP signaling and modulate Treg function, survival, and differentiation.

P2X receptors respond to extracellular ATP and include seven isoforms (P2×1R-P2×7R). P2×7R is extensively investigated for its impact on Treg stability and function. ATP binding to P2×7R induces ion influx (Ca^2+^ ,Na^+^ , and K^+^), reducing Treg suppressive capacity [13]. The agonist BzATP upregulates P2×7R expression, decreases Foxp3 levels, and promotes Th17 differentiation. In contrast, pharmacological antagonism of P2×7R promotes the conversion of naïve CD4^+^ T cells into Tregs following TCR stimulation [13]. Studies using *P2rx7*^−/−^ mice or pharmacological inhibitors such as oxidized ATP demonstrate that P2×7R expression negatively correlates with Treg function and stability [17, 54–56]. ATP-P2×7R signaling enhances Treg susceptibility to cell death at inflammatory sites, reducing their immunosuppressive function [57]. NAD^+^, released during cell damage or inflammation, activates P2×7R through ADP-ribosylation, which increases cell sensitivity to damage. Because P2×7R is more highly expressed in Tregs than in conventional T cells, NAD^+^ selectively depletes 75%–80% of Tregs [58]. Systemic NAD^+^ administration promotes an antitumor response in several mouse tumor models, suggesting a direct effect of P2×7R signaling in Treg stability and function. Another study demonstrates that NAD^+^ promotes Treg conversion into Th17 cells through purinergic signaling and activation of the transcription factors STAT3 and ROR*γ*t [59]. Culturing CD4^+^CD25^+^ Tregs with anti-CD3, anti-CD28, IL-2, and increasing concentrations of NAD^+^ significantly increases IL-17A^+^ cells, an effect attenuated in STAT3^−/−^ Tregs. NAD^+^ stimulation upregulates P2rX4 and P2rX7 mRNA expression and enhances their cell surface localization and clustering. Selective inhibition of P2×4R and/or P2×7R reduces IL-17A production and blocks Treg-to-Th17 conversion.

P2Y receptors comprise eight subtypes: P2Y1R, P2Y2R, P2Y4R, P2Y6R, P2Y11R, P2Y12R, P2Y13R, and P2Y14R. These receptors bind to various nucleotides, such as ATP, ADP, UTP, UDP, and UDP-sugars, influencing intracellular cAMP and Ca^2+^ levels. In a graft-versus-host disease (GVHD) model, P2Y2R-deficient Tregs reduced suppressive activity and impaired ability to prevent GVHD and enhance survival compared with control Tregs [60]. A recent report suggests that P2Y12R influences Treg proliferation and migration, as P2Y12R antagonism disrupts platelet-Treg interactions, reducing Treg expansion in a murine sepsis model [61].

Together, purinergic receptors and their downstream signaling pathways are critical for regulating Treg stability, metabolism, and suppressive function (Figure 2); accordingly, a range of agonists and antagonists targeting purinergic receptors or key enzymes have been developed to modulate Treg function, as summarized in Table 2.

### 1.6 | Purine Metabolic Enzymes Impact Treg Function

Purine metabolic enzymes regulate Treg stability and function. Dysregulation of these enzymes influences proinflammatory and immunosuppressive signaling, affecting Treg proliferation, migration, and suppression.

#### 1.6.1 | Extracellular Enzymes in Tregs

Elevated eATP, triggered by cell necrosis, injury, hypoxia, and cancer, significantly influences cell metabolism, adhesion, and migration during inflammation.

Tregs express ecto-enzymes CD39 and CD73, which sequentially degrade eATP into eADO, modulating purine receptor signaling [62]. CD39 (also known as ectonucleoside triphosphate diphosphohydrolase-1; ecto-NTPDase1) hydrolyzes eATP/ADP to AMP, which CD73 (also known as ecto-5’-nucleotidase) further converts to ADO, enabling Tregs to counteract ATP-induced toxicity and dendritic cell maturation via P2 receptors [63]. In humans, CD39 expression in Foxp3^+^ Tregs, regulated by TCR engagement, is essential for their immunosuppressive function, with reduced numbers of CD39+ Treg linked to autoimmune diseases like multiple sclerosis (MS) [63]. Numerous studies confirm high CD39 expression in Tregs and its pivotal role in immunosuppression [18, 64, 65]. CD73, converting AMP into ADO, plays a crucial role in Treg-mediated suppression [66, 67], demonstrated in CD73-deficient mice where its absence impairs immune tolerance in contact hypersensitivity and kidney transplant models [68, 69]. Inhibiting both CD39 and CD73 not only impedes tumor growth and metastasis but also diminishes the immunosuppressive function of Tregs and macrophages [46]. Consequently, there is significant interest in developing CD73 inhibitors due to their ability to produce ADO and induce immunosuppressive effects [70, 71].

CD38, CD157, and CD203a are cell surface ectoenzymes that regulate the extracellular nucleotide pool, particularly by producing adenosine. These enzymes also mediate noncanonical NAD^+^ signaling, influencing Treg activity [72]. CD38 converts NAD^+^ into calcium-mobilizing second messengers like cADPR, essential for Treg migration, stability, and suppression [73]. Anti-CD38 therapies, such as isatuximab, reduce CD38^+^ Tregs [74] and enhance mRNA-COVID-19 vaccine responses [75]. CD157, a paralogue of CD38 and an NAD glycohydrolase, hydrolyzes NAD^+^ into nicotinamide and ADP-ribose (ADPR), contributing to the production of ADO, which supports Treg survival and stability. CD203a hydrolyzes extracellular nucleotides into AMP and inorganic pyrophosphate (PPi) and, along with CD39 and CD73, contributes to eADO production [73], enhancing Treg-mediated immunosuppression and promoting tumor immune evasion [76].

#### 1.6.2 | Purine Salvage Pathway Enzymes

Purine salvage enzymes recycle purine bases, such as guanine, hypoxanthine, and adenine, into GMP, IMP, and AMP, maintaining nucleotide pools essential for Treg function. ADA exists as two isoenzymes, ADA1 and ADA2, each with distinct roles. ADA1, abundant in T, B, and NK cells, regulates ADO levels primarily through cytosol activity and interaction with CD26 and ADO receptors, and is essential for Treg differentiation and Foxp3 expression [77, 78]. In contrast, ADA2, predominantly expressed in myeloid cells, binds to selectively CD39^+^ Tregs but is not associated with CD26, modulates Tregs function without affecting other immune cells [79]. Hypoxanthine guanine phosphoribosyl transferase (HPRT) salvages hypoxanthine and guanine to IMP and GMP, sustaining intracellular purine pools. HPRT deficiency, as seen in Lesch-Nyhan syndrome, impairs Treg function [80], highlighting its importance in Treg-mediated immune regulation.

#### 1.6.3 | De Novo Purine Synthesis Pathway Enzymes

De novo synthesis of purines utilizes phosphoribosyl diphosphate (PRPP) from glucose metabolism to produce IMP, which is converted into AMP or GMP by key enzymes. IMP dehydrogenase (IMPDH) catalyzes the conversion of IMP to xanthosine monophosphate (XMP) utilizing NAD (Figure 1). The IMPDH inhibitor mycophenolate mofetil boosts Treg expansion, function, co-inhibitory receptor expression, and immune tolerance in transplant patients [81, 82], although direct mechanistic links between IMPDH and Treg activity are limited. Adenylosuccinate synthetase (ADSS) converts IMP to AMP through adenylosuccinate intermediates. ADSS defects can be lethal and are linked to the regulation of Foxp3 expression in iTregs [83], suggesting a potential role in Treg function, though its specific mechanisms remain uncharacterized.

#### 1.6.4 | Purine Degradation Enzymes

Uric acid (UA) is the end product of purine catabolism, with purine nucleoside phosphorylase (PNP) and xanthine oxidase (XO) as key enzymes influencing Treg function. PNP catalyzes the breakdown of purine nucleosides into purine bases and ribose-1-phosphate, regulating ADO levels. PNP deficiency in humans causes T cell immunodeficiency and autoimmune phenotypes [84]. Research suggests PNP as a metabolic immune checkpoint, influencing SAMHD1 expression during T cell development [85], promoting germinal center formation, cell cycle arrest, and activation of the interferon pathway [86], with potential association with Treg function. XO converts hypoxanthine to xanthine, and then to UA. XO-derived ROS enhance T cell proliferation [87] but impair Treg function [88], potentially leading to immune disturbances. While these enzymes influence Treg function through control of purine metabolites and byproducts like ROS, specific mechanisms remain to be fully elucidated.

### 1.7 | Differences in Purinergic Molecules Between Murine and Humans

Although there is significant therapeutic interest in purinergic molecules for various pathologic conditions, understanding their differential expression and regulation across species is crucial when translating findings from murine to human disease. For example, CD73 deficiency yields no overt pathology in mice [89], whereas in humans, it leads to vascular calcification, arteriovenous tortuosity, and joint abnormalities [90, 91]. CD39-null mice exhibit impaired Treg function and fail to prevent allograft rejection [18]. The coordinated expression of CD39/CD73 on Treg cells and the A2A receptor on activated T effector cells is required for effective immunosuppressive function. In humans, purinergic receptor expression is more complex. CD4^+^ T cells from rheumatoid arthritis patients in remission upregulate additional receptors, such as A2BR and A3R, suggesting distinct regulatory mechanisms not mirrored in mice. Ligand-binding characteristics also vary; human P2×4 receptors have higher ATP affinity (747 nM) than mice (565 nM) [92], and the antagonist 5-BDBD, a potent antagonist in humans, does not inhibit murine P2×4R [92]. A3R exhibits major interspecies variation. MRS1220 binds human A3R at 0.6 nM but binds rat A3R 50,000 times less avidly (30 μM) [93, 94]. Human P2×7R has higher sensitivity to BzATP and ATP (EC_50_: 20 and 100 μM) than murine P2×7R (295 and 850 μM) [95]. The P2Y11 receptor, involved in immune modulation, is present in humans but absent in mouse and rat genomes [96], limiting the utilization of the murine model for studying this pathway.

### 1.8 | Concluding Remarks and Future Perspectives

Several drugs targeting purine metabolism have been shown to modulate Tregs and alter immune responses. These include inhibitors of purine biosynthesis and salvage pathways (methotrexate, 6-mercaptopurine [6-MP], mycophenolate mofetil [MMF], and azathioprine [AZA]) as well as modulators of the ATP-adenosine axis (POM-1, APCP, SCH58261, and CPI-444 [ciforadenant]) (Table 3). While these drugs are used in autoimmune diseases, transplantation, and cancer therapy, challenges such as cancer metabolism heterogeneity, chemoresistance, and toxicity highlight the need for novel therapeutics with improved specificity and safety. Methotrexate and 6-MP, widely used in autoimmunity and cancer, impair Treg proliferation through disruption of nucleotide biosynthesis, yet pose risks by compromising immune tolerance and inducing systemic toxicity. MMF and AZA, effective in transplantation, similarly reduce Treg-mediated immune suppression, highlighting a mechanistic challenge in preserving balance between immune rejection and tolerance. Novel inhibitors, such as POM-1 and APCP, target ectonucleotidases (CD39 and CD73), reducing adenosine availability and suppressive signaling, with potential utility in reversing tumor-associated Treg dominance. A2A receptor antagonists, including SCH58261 and CPI-444, are under clinical evaluation to boost effector responses by dampening adenosine-driven Treg activity.

Although lack of specificity and off-target effects remain major limitations, emerging bioengineering strategies could help overcome these challenges by enabling precise delivery of otherwise nonspecific drugs to targeted cells or tissues. For example, nanoparticle-mediated delivery of small-molecule inhibitors, such as POM-1 or CPI-444, decorated with Treg- or tumor-targeting ligands could enhance local drug concentration while reducing systemic exposure [97]. Antibody–drug conjugates (ADCs) linking purine metabolism inhibitors to monoclonal antibodies specific for Treg-associated surface markers, such as CD25 or CTLA-4, could selectively deplete or modulate Tregs in pathological sites [98]. Liposome-encapsulated formulations of agents like methotrexate or MMF could facilitate preferential uptake in inflamed tissues or tumors [99] or engineered extracellular vesicles or exosomes loaded with A2A antagonists and modified with targeting peptides could direct their uptake by specific immune cell subsets or within defined anatomical niches [100] and offer the potential to enhance therapeutic precision, minimize off-target effects, and reduce systemic toxicity, thereby improving the safety and efficacy of purine metabolism–targeting strategies. Future translation requires precision approaches to delineate context-specific Treg responses, comprehensive human Treg subset profiling, and optimized dosing to balance efficacy and immune-related adverse events. Integrating metabolic profiling with immunophenotyping in human trials will be essential to unlock the therapeutic potential of purine metabolism-targeting strategies in Treg-centric diseases.

Despite accumulating evidence highlighting the critical role of purinergic signaling in Tregs and growing interest in targeting Tregs through purinergic pathways, a frequently overlooked issue is the lack of a precise definition of Treg subsets, specifically thymus-derived Tregs (tTregs), peripheral Tregs (pTregs), and induced Tregs (iTregs). These subsets differ fundamentally in origin, epigenetic programming, and functional stability [101]. tTregs develop in the thymus in response to self-antigens and exhibit stable lineage identity, characterized by complete demethylation of the FOXP3 TSDR and sustained suppressive function even under inflammatory conditions [2, 3, 33]. pTregs are generated from naive T cells in peripheral tissues in response to tolerogenic signals such as TGF-*β* and IL-2, but their epigenetic and functional stability can vary depending on environmental cues [3, 34]. In contrast, iTregs, induced in vitro under similar cytokine conditions, often display incomplete TSDR demethylation and are prone to phenotypic instability in vivo, especially in metabolically stressed or inflammatory settings [2, 102]. However, many studies on purine metabolism in Tregs, including those focused on CD39/CD73 expression [4], adenosine receptor signaling [6], and extracellular ATP sensing [9], do not distinguish among these subsets. To improve clarity and translational relevance, future studies should incorporate rigorous methods such as TSDR methylation profiling, in vivo lineage tracing, and stress-adaptive suppression assays to accurately define Treg identity when evaluating purinergic signaling.

Translating findings from animal studies to clinical applications faces challenges due to species-specific differences in purine metabolism, gene expression variations between mice and humans, and the pleiotropic effects of purinergic signaling inhibitors. Off-target effects in nonimmune cells present significant hurdles. Small-molecule inhibitors with enhanced specificity, biologics targeting purinergic enzymes or receptors, and drug delivery systems to improve efficacy while minimizing systemic toxicity may overcome these challenges. Future translational efforts should focus on cell- or tissue-specific drug delivery strategies to enhance efficacy while reducing adverse effects.

## Figures and Tables

**TABLE 1:** Context-dependent roles of extracellular ATP in Treg stability and function.

Experimental Condition/Microenvironment	Effect of eATP on Tregs	Receptors and mechanisms involved	Tissue-specific interpretation	Implications for therapy/translation	Reference
In vitro Treg generation from naïve CD4^+^ T cells	Inhibits Treg generation	P2×7 activation → reduced Foxp3 expression	Inflammatory milieu with high eATP impairs de novo Treg induction	Avoid P2×7 activation in Treg-inducing therapies	[13]
Co-culture of activated CD4^+^ T cells and Tregs	Inhibits Treg function and activates effector T cells	P2X receptor-mediated signaling	eATP acts as a danger signal, destabilizing Treg suppression in inflamed conditions	Caution in inflammatory tissues with high eATP; targeting P2 receptors may restore Treg balance	[17]
Murine gut, lamina propria under ATP exposure	Promotes Th17 over Treg development	ATP from microbiota → DC activation → IL-6 → Th17	ATP skews T cell differentiation toward inflammation in the gut	Balancing eATP and adenosine is crucial in gut inflammation therapy	[14]
Obese adipose tissue with chronic inflammation	Reduces Treg numbers and promotes Th17,	ATP signaling → proinflammatory cytokines	Metabolically stressed environments shift the Treg/Th17 balance	Targeting ATP degradation (e.g., CD39 upregulation) could restore Treg control in obesity	[15]
Tumor microenvironment postchemotherapy	Promotes Treg expansion and tolerance	DAMP + ATP → tolerogenic DCs → increased Tregs	Cell death-associated ATP release induces compensatory immunosuppression	Consider timing of immunotherapy after chemotherapy to avoid Treg-mediated tolerance	[16]

**TABLE 2:** List of agonists and antagonists targeting purine metabolic enzymes and receptors.

Enzymes	Name	Agonists	Antagonists
	CD39	ATP, ADP	POM1, ARL 67156 trisodium salt, PSB069
	CD73	AMP	AMPCP, PSB12379
	CD38	NAD+	78c
	ADK		ABT702 hydrochloride, 5-Iodotubercidin, AK-IN-1, GP3269, 8-azaadenosine, A-134974
	ADA		Pentostatin (Deoxycoformycin), 1-Deazaadenosine, EHNA hydrochloride
	IMPDH		Mycophenolate mofetil, mycophenolic acid, BMS 566419, ribavirin
	PNP		9-Deazaguanine, forodesine, ulodesine, immucillin-G
	XO		Allopurinol, febuxostat
Receptor	Subtypes		
P1	A1	Adenosine, 2-Chloro-N6-cyclopentyladenosine, N6-cyclopentyladenosine, 2’-MeCCPA, SDZ WAG 994	Caffeine, 8-cyclopentyl-1,3-dimethylaxanthine, DPCPS, KW3902
	A2A	Adenosine, CGS 21680 hydrochloride, PSB 0777 ammonium salt	Caffeine, ANR94, istradefylline, SCH442416, SCH58261, TC-G1004, ZM241385, ANR94,
	A2B	Adenosine, BAY 60–6583, LUF 5834	GS6201, MRS1754, PSB1788, PSB1115, PSB603
	A3	Adenosine, 2-Cl-IB-MECA, HEMADO, IB-MECA	DPTN, MRS1220, MRS1334, PSB10 hycrochloride
P2X	P2×1	ATP,2-MeSATP, ADP*β*S, ATP*γ*S	Suramin, PPADS, TNP-ATP, NF449, NF279, NF023
	P2×2	ATP,2-MeSATP	Suramin, PPADS
	P2×3	ATP,2-MeSATP, *αβ*-meATP	Suramin, A317491, PPADS, AF-219, gefapixan, eliapixant, TNP-ATP, NF110, A 317491 sodium salt
	P2×4	ATP, UTP, UDP	BX430, 5-BDBD, BAY1797
	P2×5	ATP, ATP*γ*S, APCPP, BzATP, ADP*β*S.	A-317491, PPADS (pyridoxal phosphate-6-azophenyl-2’,4’-disulphonic acid), suramin
	P2×6	ATP, APCPP, ATP*γ*S, BzATP	A-317491, Brilliant Blue G
	P2×7	BzATP	JNJ47965567, AZ1645373, AZ606120 dihydrochloride, A804598, A740003, A438079 hydrochloride
P2Y	P2Y1	ATP, ADP, 2-MeSATP, 2-MeSADP, ADP*β*S, MRS2365	AR-C69931MX, 2-(phenoxyaryl)-3-urea, MRS2179 tetrasodium salt, MRS2279, MRS2500, clopidogrel, ticagrelor, ticlopidine
	P2Y2	ATP, UTP, MRS2768, Diquafosol tetrasodium,	AR-C 118925XX, MRS2279
	P2Y4	ATP, UTP, UDP, 2-thio-UTP	Reactive Blue 2
	P2Y6	UTP, UDP, ADP, UDP-Glucose, MRS2693	MRS2578, TIM-38, MRS2578
	P2Y11	ATP, APCPP, BzATP, ATP*γ*S, AR-C67085	MRS2578, MRS2211, A3P5PS, NF157, suramin, PPAD
	P2Y12	ATP, ADP, 2-MeSADP, 2-MeSATP, ADP*β*S)	AR-C66096, Tetrasodium salt, AR-C69931, clopidogrel, ticlopidine, ticagrelor, prasugrel, cangrelor, AZD1283, WSJ-557, PSB0739
	P2Y13	ADP, ADP*α*S, 2-MeSADP, 2-MeSATP, ADP-*β*-S	MRS2211, MRS2578, A3P5PS, suramin, PPADS
	P2Y14	UTP, UDP, UDP-glucose, UDP-glucuronate, 2-Thio-UDP	AZ10606120, PPTN, MRS2578, suramin, PPADS

Abbreviations: ATP*γ*S, adenosine 5’-[*γ*-thio]triphosphate; BzATP, (2’(3’)-O-(4-benzoylbenzoyl)ATP); ATP*γ*S, adenosine 5’-O-(3-thiotriphosphate); 2-MeSADP, 2-methylthio-ADP; 2-MeSATP, 2-methylthio-ATP; 2-Thio-UDP, 2-thio-uridine 5’-diphosphate; ADP*β*S, adenosine 5’-O-(2-thiodiphosphate); APCPP, adenosine 5’-[*α*,*β*-methylene]triphosphate]; ADP*β*S, adenosine 5’-O-(2-thiodiphosphate).

**TABLE 3:** Drugs targeting purine metabolism, effects on Treg function, and clinical relevance.

Inhibitor	Target	Mechanisms	Impact on Tregs and disease progression	Clinical relevance	Limitations	Therapeutic potential and future directions
Methotrexate (MTX)	Dihydrofolate reductase (DHFR)	Inhibits purine synthesis by blocking folate metabolism	Reduces Treg proliferation and function, promotes inflammation [102, 103]	Used in rheumatoid arthritis and cancer therapy, it can exacerbate autoimmunity	Hepatotoxicity, bone marrow suppression, pulmonary toxicity; promotes inflammation due to Treg suppression [104]	Widely used anti-inflammatory; careful dose modulation may preserve Tregs; future potential in combination with Treg-sparing agents
6-Mercaptopurine (6-MP)	Hypoxanthine-guanine phosphoribosyltransferase (HGPRT)	Inhibits purine nucleotide synthesis, disrupting ATP/GTP pools	Impairs Treg function, promotes immune activation in autoimmunity [105]	Used in acute lymphoblastic leukemia and IBD; may disrupt immune tolerance	Hepatotoxicity, myelosuppression, increased susceptibility to infection [106]	Refinement of dosing or delivery systems may enhance efficacy while preserving Tregs in autoimmune contexts
Mycophenolate mofetil (MMF)	Inosine monophosphate dehydrogenase (IMPDH)	Blocks de novo guanine nucleotide synthesis	Reduces Treg proliferation, promotes immune rejection in transplantation [107]	Used in organ transplantation to prevent rejection; limits Treg-mediated immune suppression	Gastrointestinal disturbances, leukopenia, increased risk of infection [108]	Future strategy may involve selective Treg-sparing IMPDH inhibitors or Treg supplementation
Azathioprine (AZA)	Purine biosynthesis	Incorporates into DNA, disrupting purine metabolism	Alters Treg homeostasis, enhances effector T cell responses [109]	Used in autoimmune diseases (lupus, IBD) and transplant rejection prevention	Hepatoxicity, bone marrow suppression, increased risk of infection [110]	Combination with Treg-promoting cytokines may offset immunosuppressive toxicity
POM-1	CD39	Inhibits ATP hydrolysis to AMP	Reduces adenosine production, limits Treg-mediated suppression [111]	Under investigation for cancer immunotherapy by reducing Treg-mediated immune suppression	Not yet approved for clinical use; risks of off-target effects and systemic toxicity [112]	Promising in tumors with high CD39+ Tregs; needs targeting strategies (e.g., tumor-specific delivery)
APCP	CD73	Inhibits AMP hydrolysis to adenosine	Decreases extracellular adenosine, impairs Treg function [66]	Potential anti-tumor agent by preventing adenosine-mediated immunosuppression	Limited clinical data available; risks of off-target effects and systemic toxicity [113]	May synergize with immune checkpoint inhibitors in adenosine-rich tumors
SCH58261	A2A adenosine receptor	Blocks adenosine signaling	Reduces Treg-mediated immune suppression, enhances inflammation [114]	Investigated for cancer immunotherapy to enhance anti-tumor immunity and as a potential therapy for Alzheimer’s disease	Not yet approved for clinical use; possible central nervous system-related side effects [114]	Potential in tumors with high adenosine; warrants tissue-specific or controlled-release formulations
CPI-444 (Ciforadenant)	A2A adenosine receptor	Inhibits adenosine signaling in the tumor microenvironment	Decreases Treg function, enhances effector T cell responses [115]	In clinical trials for solid tumors to improve immune response	Clinical efficacy and safety profiles still under investigation [116]	Promising as part of combination immunotherapy, may benefit from patient stratification by adenosine levels

## Data Availability

Data sharing is not applicable to this article as no datasets were generated or analyzed during the current study.

## Abbreviations:

A2AR: adenosine A2A receptor
A2BR: adenosine A2B receptor
ADO: adenosine
ATP: adenosine triphosphate
Camp: cyclic adenosine monophosphate
eADO: extracellular adenosine
eATP: extracellular adenosine triphosphate
FAO: fatty acid oxidation
Foxp3: Forkhead box protein P3
GPCRs: G protein-coupled receptors
IL-10: interleukin-10
OXPHOS: oxidative phosphorylation
P2×7R: P2×7 receptor
P2Y2R: P2Y2 receptor
POM-1: sodium polyoxotungstate
Teffs: effector T cells
TGF-*β*: transforming growth factor-beta
TME: tumor microenvironment
Tregs: regulatory T cells
